# The Moderating Effect of Suggestibility on the Relationship between Body Mass Index and Body Dissatisfaction in Women

**DOI:** 10.3390/jcm13164647

**Published:** 2024-08-08

**Authors:** Franck-Alexandre Meschberger-Annweiler, Mariarca Ascione, Bruno Porras-Garcia, Maria Teresa Mendoza-Medialdea, Marta Ferrer-Garcia, Jose Gutierrez-Maldonado

**Affiliations:** 1 Department of Clinical Psychology and Psychobiology, Facultat de Psicologia, Universitat de Barcelona (UB), Passeig de la Vall d’Hebron 171, 08035 Barcelona, Spain; franck.meschberger@ub.edu (F.-A.M.-A.); ascione.m@ub.edu (M.A.); tmendoza@ub.edu (M.T.M.-M.); martaferrerg@ub.edu (M.F.-G.); 2 Institut de Neurociences (UBneuro), Universitat de Barcelona (UB), Passeig de la Vall d’Hebron 171, 08035 Barcelona, Spain; 3 Brain, Cognition and Behavior Research Group, Consorci Sanitari de Terrassa (CST), 08221 Terrassa, Spain; bporras@uic.es; 4 Department of Basic Sciences, Universitat Internacional de Catalunya, 08195 Sant Cugat del Valles, Spain; 5 Department of Psychology, Universidad de Jaén, Paraje las Lagunillas s/n, 23071 Jaén, Spain

**Keywords:** body dissatisfaction, body mass index, suggestibility, influenceability, eating disorders

## Abstract

**Background**: Body dissatisfaction (BD) has been consistently linked to adverse consequences on mental health and overall well-being, and is recognized as a significant contributing factor in the initiation and persistence of eating disorders (EDs). Empirical evidence has demonstrated that an elevated body mass index (BMI) and media influence and pressure about a thin ideal heighten the risk of subsequent BD. Moreover, suggestibility, a propensity to accept and act upon messages without critical evaluation, has been shown to be positively associated with greater susceptibility to the influence of sociocultural messages that endorse the thin ideal. This study aimed to assess whether suggestibility moderates the association between BMI and BD in women. **Methods**: A total of 117 women completed assessments using the Eating Disorder Inventory-3 (EDI-3) BD subscale and the Suggestibility Inventory, which encompasses a general suggestibility index and a subscale that evaluates susceptibility to influence by others. We conducted moderation analyses employing the PROCESS macro, with BMI as the central predictor, BD as the outcome variable, and suggestibility and its subscale as moderators. **Results**: The findings revealed statistically significant positive moderating interactions for both the general suggestibility index and susceptibility to influence by others. Specifically, women who exhibited high levels of suggestibility and susceptibility to influence by others demonstrated a more pronounced increase in BD as their BMI increased. **Conclusions:** These outcomes are in line with the sociocultural model of EDs, suggesting that greater susceptibility to external influences amplifies the impact of societal pressures to conform to thin ideals.

## 1. Introduction

Body dissatisfaction is a prevalent concern among adolescents and young adults, with reported prevalence rates ranging from 24% to 46% among adolescent girls [1]. Previous research consistently demonstrates that women experience higher rates of body dissatisfaction compared to men [2,3,4]. For instance, a recent study by Rosenqvist et al. (2023) [5] reported overall prevalence rates of body dissatisfaction ranging from 14% to 22% in women and 8% to 12% in men. This negative subjective evaluation of one’s own body has been consistently linked to a wide array of adverse outcomes on mental health and overall well-being. It has been shown to predict low self-esteem [6], the onset of depressive symptoms [7], and even suicidal ideation [8]. Additionally, body dissatisfaction has been associated with a decline in subjective quality of life [9]. Furthermore, body dissatisfaction represents the affective component of body image disturbances, which is a core diagnostic criterion for anorexia nervosa and bulimia nervosa, as outlined in the *Diagnostic and Statistical Manual of Mental Disorders* (fifth edition, DSM-5) [10]. Numerous studies (as reviewed by Shagar et al., 2017 [11]) have underscored its significance as a risk factor for the emergence of eating disorder (ED) symptoms among adolescents and young adults. Body dissatisfaction’s association with weight-related behaviors, such as abnormal eating attitudes, dieting practices, extreme weight-control behaviors, weight-altering methods, eating attitudes, weight-control attempts and strategies, and dietary habits, further underscores its pivotal role in the development of EDs. Moreover, body dissatisfaction has been identified as a significant predictor of anorexia readiness syndrome [12], a cluster of cognitive, emotional, and behavioral indicators related to challenges in meeting nutritional needs and one’s attitudes toward their body [13]. It also serves as a predictor for diagnosed EDs [14,15,16] and ED symptoms, such as binge eating, purging, restriction, excessive exercise, and muscle-building behaviors [17]. Such predictions may account for 16% and 23% of the explained variance in anorexia nervosa (AN) and bulimia nervosa (BN) symptomatology, respectively [18]. Additionally, other studies have demonstrated that body dissatisfaction increases the risk of ED relapse [19,20].

Numerous studies have concentrated on identifying the potential predictors of body dissatisfaction. Body mass index (BMI) has been shown to be highly correlated with body dissatisfaction, even when controlling for other factors (e.g., [14,21,22]). However, longitudinal research is needed to track the evolution of this relationship over time. For instance, a recent longitudinal study by Blundell et al. (2024) [23] demonstrated a clear trajectory from higher childhood BMI to increased body dissatisfaction and subsequent depressive symptoms in adolescence, with these effects being twice as large in girls as in boys. Numerous studies have consistently demonstrated this gender disparity. For example, the meta-analysis by Weinberger et al. (2017) [24] showed that women with obesity were more dissatisfied with their bodies than men with obesity compared to their respective normal-weight peers, and that women reported significantly higher body dissatisfaction even if their BMIs were lower than those of men.

However, current research highlights the need for a nuanced understanding of the relationship between BMI and body dissatisfaction. Factors such as body composition, personality, cultural background, and socioeconomic status should also be considered as influencers in this association. In addition, caution is warranted when interpreting gender differences in the BMI–body dissatisfaction relationship, as the underlying mechanisms remain complex and are still under investigation. Societal pressures, cultural norms, and media representations are widely also acknowledged as significant contributors to body dissatisfaction in women. Among the variables related to media influence, the internalization of the media’s thin ideal, and perceived pressure from the media, significantly heighten the risk of subsequent body dissatisfaction in women [14,25]. The thin ideal is a cultural concept that emphasizes an unrealistically slim body type, particularly for women, that emphasizes low body fat (e.g., a very slender physique with minimal visible fat), minimal curves (e.g., a flat stomach, a lack of any visible abdominal definition), and a focus on thinness (e.g., a significantly narrower waist compared to the hips and shoulders) as a marker of beauty and social value [26]. The thin ideal, propagated by the media (e.g., [27]), delineates the societal standard of attractiveness within a particular culture and profoundly impacts how young individuals perceive and evaluate themselves and their bodies through social comparisons [28]. Often unattainable, this thin ideal creates a dissonance with one’s actual body (e.g., disparities between actual and ‘thin ideal’ BMIs), resulting in body-related concerns and body dissatisfaction [29]. This mechanism has been substantiated by numerous studies that have established significant associations between the thin ideal, the use of social media, and body dissatisfaction (e.g., [30,31]).

In our contemporary sociocultural milieu, where marketing and social media inundate individuals with messages related to unhealthy foods and weight-loss products (e.g., see [32,33]), suggestibility, defined as a personality trait characterized by a propensity to accept and act upon messages without critical evaluation [34], may thus assume particular relevance in the underlying mechanisms that influence body dissatisfaction. For example, suggestibility has been shown to be a predictor of thin ideal internalization [35]. Furthermore, previous studies have shown that higher levels of suggestibility have been negatively associated with a positive body image [36], and positively linked to greater susceptibility to the influence of sociocultural messages that endorse the thin ideal (e.g., [37]) and to the consumption of palatable foods for reward, social, and conformity reasons; active dieting; and binge-eating [38]. While limited research directly examines the relationship between BMI and suggestibility, previous studies have not found any significant correlation between these variables [38], nor between suggestibility and obesity [39].

As a result, the present study aims to contribute to a better understanding of the impact of suggestibility on body dissatisfaction. Building on the established knowledge that women experience higher rates of body dissatisfaction and eating disorders (EDs) compared to men (as detailed above), this study specifically aims to investigate whether suggestibility moderates the relationship between BMI and body dissatisfaction in a sample of healthy female participants, bringing the first evidence that may orientate future research. Our objective is twofold: (1) to enhance body-related well-being in women—by exploring the role of suggestibility, we hope to identify factors that influence body dissatisfaction and inform interventions aimed at promoting a positive body image in women; (2) to improve ED prevention and treatment for female patients—understanding how suggestibility interacts with body dissatisfaction can provide valuable insights for developing more effective ED prevention campaigns and treatment strategies specifically tailored to women. It is hypothesized that healthy women who exhibit high levels of suggestibility will demonstrate a more pronounced increase in body dissatisfaction as their BMI increases.

## 2. Materials and Methods

### 2.1. Participants

This study was approved by the ethics committee of the University of Barcelona. The minimum sample size was initially determined using a statistical power of 0.8, a significance level of 0.05, and a Pearson correlation of 0.43 between BMI and body dissatisfaction, as reported in a prior study [21]. The required sample size was determined to be at least N = 40. We recruited a total of 122 healthy female college students from the University of Barcelona using social networks and flyers to voluntarily participate in this study. Before the start of the study, each participant freely signed a consent form. The exclusion criteria were self-reported diagnoses of ED (AN, BN, binge ED), self-reported diagnoses of mental disorders with psychotic or manic symptoms (e.g., psychotic disorders or bipolar disorders), and pregnancy (which could temporarily distort body image perception and self-evaluation). Five volunteers were excluded from the study as they reported meeting at least one of the exclusion criteria. Ultimately, 117 healthy female college students participated in the study and completed the entire procedure (M_age_ = 24.21 years, SD_age_ = 5.36 years, M_BMI_ = 22.45 kg/m^2^, SD_BMI_ = 3.16 kg/m^2^).

### 2.2. Measures

Body mass index was calculated after measuring the participant’s weight and height on-site, using the formula: BMI = weight (in kg)/ height (in m)^2^.

Body dissatisfaction was evaluated using the Spanish version of the Eating Disorder Inventory-3 (EDI-3), which was translated by Elosua et al. (2010) [40] from the original instrument developed by Garner (2004) [41]. The EDI-3 is a self-report inventory comprising 12 scales and a total of 91 items, with responses provided on a 6-point Likert scale. In this study, we exclusively utilized the 10-item Body Dissatisfaction scale (EDI-BD) from the EDI-3, which assesses body dissatisfaction encompassing both overall body perception and specific body parts. The EDI-BD questionnaire employed for data collection in the present study is available in Supplementary Materials “S1—EDI-3 Body Dissatisfaction questionnaire”. The Spanish version of this scale has demonstrated robust psychometric properties, including strong validity indices, temporal stability (with a test–retest reliability of *r* = 0.86), and excellent internal consistency (with Cronbach’s alpha values ranging from 0.74 to 0.96) [40,42]. In the current study, the internal consistency (Cronbach’s *α*) for the EDI-BD scale was 0.85.

Suggestibility was assessed using the Suggestibility Inventory [43], a self-reported 22-item inventory rated on a 5-point Likert scale that provides a general index of suggestibility (Sugg_Gen) distributed across four factors: dreaming/fantasizing, absorption, emotional involvement, and influence by others. Dreaming/fantasizing describes the subject’s tendency to escape or get carried away through imagery, music, or voice, i.e., reveals the individual’s ability to fantasize about things or “daydream”. Absorption describes the subjects’ ability to focus their attention, concentrate on their own images and sensations, or vividly experience sensations through the imagination. Emotional involvement refers to the individual’s capacity to become emotionally and actively engaged with the content of the prompted message. Influence by others describes the degree to which individuals allow others to influence their attitudes, thoughts, and state of mind. The translation to English from the original Spanish version of the Suggestibility Inventory employed in the present study is available in Supplementary Materials “S2—Suggestibility Inventory”. The author of the scale, based on a sample of over 600 participants, reported a high level of internal consistency (*α*_Cronbach_ = 0.79), and good stability over time, with a 3-month test–retest reliability coefficient of 0.70 for the general index of suggestibility [43]. However, the emotional involvement (*r*_test–retest_ = 0.81) and influence by others (*r*_test–retest_ = 0.71) factors showed better stability over time than the dreaming/fantasizing and absorption factors (*r*_test–retest_ = 0.55 and 0.49, respectively, which cannot be considered as acceptable). In the present study, the internal consistency (Cronbach’s α) was calculated for the general index of suggestibility and its four subscales, with the following results: general index of suggestibility: *α*_Cronbach_ = 0.77 (acceptable), dreaming/fantasizing: *α*_Cronbach_ = 0.70 (acceptable), absorption: *α*_Cronbach_ = 0.47 (unacceptable), emotional involvement: *α*_Cronbach_ = 0.50 (poor), and influence by others: *α*_Cronbach_ = 0.74 (acceptable). For this reason, any results that involve specifically one of the three subscales dreaming/fantasizing, absorption or emotional involvement will have to be interpreted with caution due to concerns about their reliability and/or internal consistency.

### 2.3. Procedure

Before the start of the study, each participant freely signed a consent form, which informed the participants about the confidentiality of the data, the possibility of withdrawing from the study at any time without consequences, and explained the procedure. Confidentiality was ensured by assigning a different identification code to each participant. The participants’ weight and height were measured in order to calculate their BMI. After verifying the exclusion criteria, the participants completed the questionnaires (EDI-BD and the Suggestibility Inventory) on a computer, through the Qualtrics^XM^ platform (Qualtrics International Inc., Provo, UT, USA), for approximately 15 min.

### 2.4. Statistical Analysis

Initially, a correlation analysis was conducted to identify any potentially significant Pearson correlation coefficients between body dissatisfaction, BMI, the general index of suggestibility, and each of its four factors (dreaming/fantasizing, absorption, emotional involvement, and influence by others).

**Figure 1:**
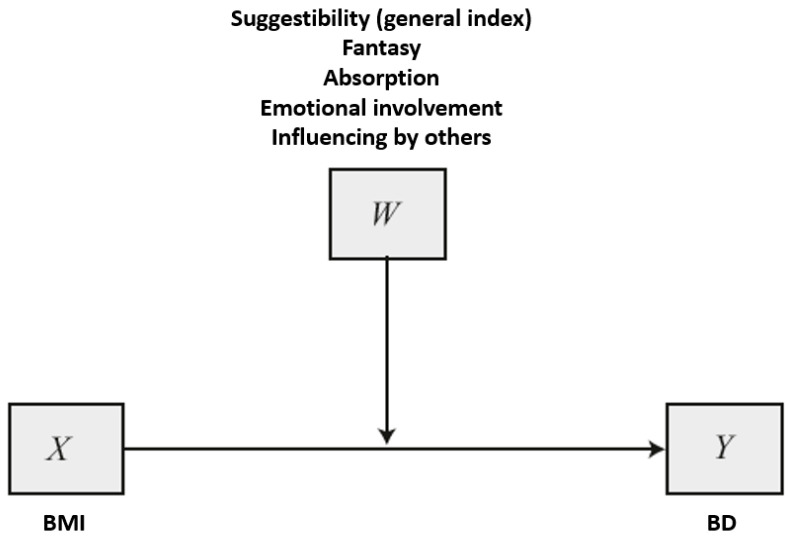
Simple moderation (PROCESS model 1). Model based on p. 621 in Hayes (2022) [44]. BMI = body mass index, BD = body dissatisfaction assessed through the body dissatisfaction scale of the Spanish version of Eating Disorder Inventory-3. General index of suggestibility, dreaming/fantasizing, absorption, emotional involvement and, influence by others were analyzed separately as moderator variable W.

Then, five moderation analyses were conducted using model 1 (simple moderation) of PROCESS macro v.4.1 [44], with BMI as a focal predictor (X), body dissatisfaction as an outcome (Y), and the general suggestibility index and each of the four suggestibility factors as moderators (W; each of them considered independently; see Figure 1). The required linear regression assumptions were verified as follows: no outliers were detected (the criterion used for outliers was ±3 SD); linear relationships among the variables were apparent using scatter plots; there was homoscedasticity of the residuals (uniform variation of the residuals with predicted values as indicated by non-significant Pearson correlation: *p* > 0.05); and there was independence of the residuals, as assessed by a Durbin–Watson statistic (between 1.5 and 2.5). The assumption of the normality of residuals was met through the use of the bootstrap technique with 5000 samples, which yields more robust estimates of standard error (see more details in Li et al., 2012 [45], and on pp. 72–73 in Hayes, 2022 [44]). In order to enable the reproducibility of the results, the bootstrapping SEED was set to 70575. Regarding non-multicollinearity, Hayes (2022) [44] indicates that the regression results remain reliable even if this assumption is not verified, in the case of moderation analysis that involves only one or two interactions, as is the case in the current study (see pp. 323–325 in Hayes, 2022 [44]). Finally, the Johnson–Neyman technique was applied to identify regions of significance within the regression models (see detailed information on pp. 269–275 in Hayes, 2022 [44]).

All the analyses were conducted with SPSS v.27 (IBM Company, Armonk, NY, USA).

## 3. Results

The descriptive statistics of the complete sample (N = 117) are given in Table 1.

Significant 2-tailed Pearson correlation coefficients were found between body dissatisfaction and, respectively, BMI (*r*_pearson_ = 0.51, *p* < 0.001), general index of suggestibility (*r*_pearson_ = 0.20, *p* = 0.03), and influence by others (*r*_pearson_ = 0.35, *p* < 0.001), but not with the other three suggestibility subscales.

**Figure 2:**
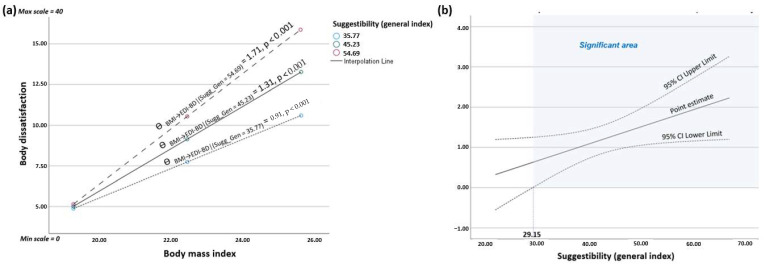
Moderation analysis of the general index of suggestibility in the relationship between body mass index and body dissatisfaction: (**a**) visualization of the conditional effects of the focal predictor (BMI) at values of the moderator “general index of suggestibility” (at mean and ±1*SD*); (**b**) significant area of the moderator “general index of suggestibility” (Johnson–Neyman method).

The moderation analysis revealed a statistically significant positive interaction effect between BMI and the general index of suggestibility on body dissatisfaction (*p* = 0.035). The analysis of the conditional effects indicated that, in women with a high general index of suggestibility (i.e., scoring Sugg_Gen = mean + 1*SD* = 54.69), the effect of BMI on body dissatisfaction (*θ* _X→Y |(W=54.69)_ = 1.71, *p* < 0.001) was higher than in women with a low general index of suggestibility (i.e., scoring Sugg_Gen = mean − 1*SD* = 35.77; *θ* _X→Y |(W=35.77)_ = 0.91, *p* < 0.001) (see Figure 2a). This model accounted for 34% of the explained variability of body dissatisfaction. Moreover, using the Johnson–Neyman technique, the critical value in the moderating variable “general index of suggestibility” from which the effect of BMI on the body dissatisfaction began to be statistically significant, was 29.15 on a scale with a theoretical range from 0 to 88 (see Figure 2b). In total, 94.87% of the study participants exceeded that threshold.

**Figure 3:**
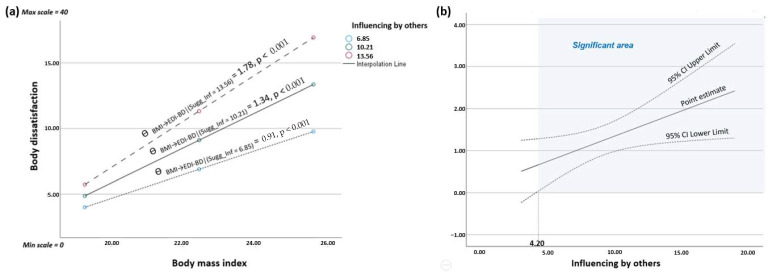
Moderation analysis of the influence by others factor on the relationship between body mass index and body dissatisfaction: (**a**) visualization of the conditional effects of the focal predictor (BMI) at values of the moderator influence by others (at mean and ±1*SD*); (**b**) significant area of the moderator influence by others (Johnson–Neyman method).

In addition, moderation analysis also showed a statistically significant positive interaction effect between BMI and the influence by others subscale of suggestibility on body dissatisfaction (*p* = 0.013). The visualization of the conditional effects of the focal predictor (BMI) at values of the moderator influence by others at mean and ±1*SD* indicated that the impact of BMI on body dissatisfaction was more pronounced in women with a high susceptibility to influence by others compared to women with a lower susceptibility to influence by others (see Figure 3a). This model accounted for 42% of the explained variability of body dissatisfaction. The Johnson–Neyman technique revealed that the critical value on the moderating variable influence by others, at which the effect of BMI on body dissatisfaction became statistically significant, was 4.20 on a scale ranging from 0 to 20 (see Figure 3b). A total of 95.72% of the study participants exceeded this critical value.

Finally, no significant moderation interaction was observed with the other three subscales of suggestibility (dreaming/fantasizing, absorption and emotional involvement) as potential moderators. However, the results for these three subscales warrant cautious interpretation due to concerns about their reliability and/or internal consistency. Specifically, study by Gonzalez-Ordi and Miguel-Tobal (1999) [43] and our own data suggest these subscales may have poor reliability or internal consistency, as indicated above.

## 4. Discussion

The results of this study provide some insights into the complex relationship between BMI, body dissatisfaction, and suggestibility in women. The results highlight the role of interpersonal factors and social influences in shaping body dissatisfaction. Both the overall index of suggestibility and its influence by others subscale were discovered to moderate the relationship between BMI and body dissatisfaction. These moderating effects attained statistical significance at extremely low scores on the Suggestibility Inventory (specifically, 29.15 on an 88-point scale for the overall index of suggestibility and 4.20 on a 20-point scale for the influence by others subscale) encompassing nearly the entire study sample. Consequently, although previous studies [38,39] found no direct correlation between suggestibility and BMI, our results indicate that suggestibility may indirectly influence the complex relationship between BMI and body dissatisfaction in healthy women by acting as a moderator. This aligns with previous research demonstrating a negative association between suggestibility and positive body image [36], and substantiates that future research should elucidate the role of suggestibility in the complex mechanisms underlying the BMI–body dissatisfaction association.

Moreover, the model integrating the influence by others factor as a moderator explained a larger proportion of the variability in body dissatisfaction compared to the model with the overall index of suggestibility as a moderator (42% vs. 34%). This implies that susceptibility to external influence plays a more significant role in the moderation process than general suggestibility itself, including all its subscales. Women who are more susceptible to external pressures, judgments, or comments from others may experience more pronounced body dissatisfaction. These findings align with the sociocultural model of eating pathologies, as higher levels of suggestibility to influence by others might magnify the impact of sociocultural pressures to conform to thin ideals [12]. This susceptibility could also contribute to individuals’ attentiveness to body-related comments from others, thus intensifying body dissatisfaction. Consequently, heightened body dissatisfaction predisposes individuals to engage in dieting, encounter negative affect, and develop eating disorders [46].

The participants in this study were young college women without diagnosed eating disorders. Future research could enhance this scope by including a more diverse range of participants, encompassing individuals clinically diagnosed with eating disorders as well as healthy participants from various cultural backgrounds. Previous research has indicated significant variations in body dissatisfaction among clinical patients diagnosed with different eating disorders, with the highest levels observed in individuals with BN and purgative AN compared to those with restrictive AN or binge ED [47]. Additionally, age and sex/gender may influence the relationship between BMI and body dissatisfaction. Prior studies have shown that body dissatisfaction was assessed, perceived, or managed differently across successive life stages: childhood, adolescence, early and mid-adulthood, and was dependent on sex/gender [2,3,4,5,48,49]. Other variables, such as race or sexual orientation, may also warrant consideration in future research, as previous studies have indicated [50,51]. Furthermore, suggestibility may be influenced by factors such as age, gender, or sex, although such associations have been more extensively studied in different contexts such as forensic settings examining interrogative suggestibility, or in the domain of hypnotherapy, rather than within the realm of body image or media influence research. Future research endeavors can consider a broader spectrum of variables, building upon those mentioned previously (e.g., age, sex, etc.). These could encompass additional variables identified in prior studies and/or theoretical models, such as thin ideal internalization [52], weight bias internalization [53], social media usage (e.g., [30,31]), personality traits (e.g., extraversion, openness, conscientiousness, neuroticism, and agreeableness), or even cognitive styles (e.g., internal/external locus of control, convergent/divergent thinking, reflectivity/impulsivity, etc.), to explore their potential roles as moderators or mediators. Finally, future research should consider employing a more psychometrically robust measure of suggestibility than the inventory by Gonzalez-Ordi and Miguel-Tobal (1999) [43], given concerns about the reliability of its subscales related to dreaming/fantasizing, absorption, and emotional involvement (as previously stated).

Although further research is needed, our results showed that suggestibility significantly moderates the relationship between BMI and body dissatisfaction in healthy young women. As body dissatisfaction might be an important modifiable target for preventive interventions aimed at reducing onset of ED and/or depressive symptoms [14,21,22,23,24], particularly among adolescent girls, knowledge of this moderation effect could potentially help inform and tailor interventions or preventive campaigns aimed at fostering positive body image, improving healthy eating habits, preventing and treating diet-related disorders, particularly in contexts involving external and social influences (such as social networks). Suggestibility should be considered when identifying and monitoring people at risk of EDs, in order to protect those that may be most susceptible to adopting invalid messages and products. In addition, by acknowledging the complexity of the association between BMI and body dissatisfaction, and by focusing on a broader range of factors such as suggestibility, researchers can also develop more effective interventions to address body dissatisfaction and promote body positivity in female patients diagnosed with EDs or other mood disorders related to negative body image. Consequently, if future research were to also show such a significant moderating effect of suggestibility on the relationship between BMI and body dissatisfaction in the clinical population, potential interventions could encompass: critical thinking skills enhancement (e.g., fostering critical thinking can help individuals question information and make independent judgments); media literacy (e.g., educating individuals about media manipulation techniques can reduce susceptibility to influence); assertiveness training (e.g., developing assertiveness skills can empower individuals to express their opinions and resist peer pressure); cognitive behavioral therapy (e.g., helping individuals identify and challenge negative thought patterns that contribute to suggestibility); mindfulness and meditation (e.g., enhancing self-awareness and reducing susceptibility to external influences).

In summary, this study introduces a moderation model that explains 42% of the variance in body dissatisfaction among college women. The recognition of suggestibility’s moderating role in the BMI–body dissatisfaction association holds significant implications for interventions aimed at mitigating body dissatisfaction and a fostering positive body image. Tailoring these interventions to account for an individual’s suggestibility level, particularly in contexts involving external influences, could potentially enhance their efficacy. Future research can study other moderation and mediation models to further enhance the predictive model for body dissatisfaction and gain a deeper understanding of the underlying mechanisms within the complex relationship between BMI and body dissatisfaction.

## Figures and Tables

**Table 1:** Descriptive statistics of the entire sample.

	Mean	Standard Deviation	Sample Range Min.–Max.	Scale Theorical Range Min.–Max.
Age	24.21	5.36	20–57	N/A
Body mass index	22.45	3.16	17.65–33.67	N/A
Body dissatisfaction (EDI-BD)	9.09	7.18	0–35	0–40
Suggestibility (general index)	45.23	9.46	22–67	0–88
Dreaming/fantasizing	8.78	3.09	2–15	0–16
Absorption	9.09	2.34	3–15	0–16
Emotional involvement	9.63	3.38	0–19	0–20
Influence by others	10.21	3.35	3–19	0–20

Note. N/A = not applicable. EDI-BD = body dissatisfaction scale of the Spanish version of the Eating Disorder Inventory-3.

## Data Availability

The data presented in this study are available on request from the corresponding author.

## Supplementary Materials

The following supporting information can be downloaded at: https://www.mdpi.com/article/10.3390/jcm13164647/s1. File S1. EDI-3 Body Dissatisfaction Questionnaire. File S2. Suggestibility Inventory.

## References

[1] Wang S.B., Haynos A.F., Wall M.M., Chen C., Eisenberg M.E., Neumark-Sztainer D. (2019). Fifteen-Year Prevalence, Trajectories, and Predictors of Body Dissatisfaction From Adolescence to Middle Adulthood. Clin. Psychol. Sci. A J. Assoc. Psychol. Sci..

[2] Quittkat H.L., Hartmann A.S., Düsing R., Buhlmann U., Vocks S. (2019). Body dissatisfaction, importance of appearance, and body appreciation in men and women over the lifespan. Front. Psych..

[3] McCabe M.P., Ricciardelli L.A. (2004). Body image dissatisfaction among males across the lifespan: A review of past literature. J. Psychosom. Res..

[4] Esnaola I., Rodríguez A., Goñi A. (2010). Body dissatisfaction and perceived sociocultural pressures: Gender and age differences. Salud Ment..

[5] Rosenqvist E., Konttinen H., Berg N., Kiviruusu O. (2023). Development of Body Dissatisfaction in Women and Men at Different Educational Levels during the Life Course. Int. J. Behav. Med..

[6] Paxton S.J., Neumark-Sztainer D., Hannan P.J., Eisenberg M.E. (2006). Body dissatisfaction prospectively predicts depressive mood and low self-esteem in adolescent girls and boys. J. Clin. Child. Adolesc. Psychol..

[7] Holsen I., Kraft P., Roysamb E. (2001). The Relationship between Body Image and Depressed Mood in Adolescence: A 5-year Longitudinal Panel Study. J. Health Psychol..

[8] Kim D.S., Kim H.S. (2009). Body-image dissatisfaction as a predictor of suicidal ideation among Korean boys and girls in different stages of adolescence: A two-year longitudinal study. J. Adolesc. Health.

[9] Mond J., Mitchison D., Latner J., Hay P., Owen C., Rodgers B. (2013). Quality of life impairment associated with body dissatisfaction in a general population sample of women. BMC Public Health.

[10] American Psychiatric Association (2013). Diagnostic and Statistical Manual of Mental Disorders.

[11] Shagar P.S., Harris N., Boddy J., Donovan C.L. (2017). The Relationship between Body Image Concerns and Weight-Related Behaviours of Adolescents and Emerging Adults: A Systematic Review. Behav. Chang..

[12] Rymarczyk K. (2021). The role of personality traits, sociocultural factors, and body dissatisfaction in anorexia readiness syndrome in women. J. Eat. Disord..

[13] Ziolkowska B., Ocalewski J., Dabrowska A. (2021). The Associations between the Anorexic Readiness Syndrome, Familism, and Body Image among Physically Active Girls. Front. Psychiatry.

[14] Stice E., Shaw H.E. (2002). Role of body dissatisfaction in the onset and maintenance of eating pathology: A synthesis of research findings. J. Psychosom. Res..

[15] Rohde P., Stice E., Marti C.N. (2015). Development and Predictive Effects of Eating Disorder Risk Factors during Adolescence: Implications for Prevention Efforts. Int. J. Eat. Disord..

[16] Mustapic J., Marcinko D., Vargek P. (2015). Eating behaviours in adolescent girls: The role of body shame and body dissatisfaction. Eat. Weight Disord..

[17] Romano K.A., Heron K.E., Henson J.M. (2021). Examining associations among weight stigma, weight bias internalization, body dissatisfaction, and eating disorder symptoms: Does weight status matter?. Body Image.

[18] Brannan M.E., Petrie T.A. (2008). Moderators of the Body Dissatisfaction-Eating Disorder Symptomatology Relationship: Replication and Extension. J. Couns. Psychol..

[19] Carter J.C., Blackmore E., Sutandar-Pinnock K., Woodside D.B. (2004). Relapse in anorexia nervosa: A survival analysis. Psychol. Med..

[20] Keel P.K., Dorer D.J., Franko D.L., Jackson S.C., Herzog D.B. (2005). Postremission predictors of relapse in women with eating disorders. Am. J. Psychiatry.

[21] Porras-Garcia B., Ferrer-Garcia M., Yilmaz L., Sen Y.O., Olszewska A., Ghita A., Serrano-Troncoso E., Treasure J., Gutiérrez-Maldonado J. (2020). Body-related attentional bias as mediator of the relationship between body mass index and body dissatisfaction. Eur. Eat. Disord. Rev..

[22] Kakeshita I.S., Almeida S.S. (2008). The relationship between body mass index and body image in Brazilian adults. Psychol. Neurosci..

[23] Blundell E., De Stavola B.L., Kellock M.D., Kelly Y., Lewis G., McMunn A., Nicholls D., Patalay P., Solmi F. (2024). Longitudinal pathways between childhood BMI, body dissatisfaction, and adolescent depression: An observational study using the UK Millennium Cohort Study. Lancet Psych..

[24] Weinberger N.A., Kersting A., Riedel-Heller S.G., Luck-Sikorski C. (2017). Body Dissatisfaction in Individuals with Obesity Compared to Normal-Weight Individuals: A Systematic Review and Meta-Analysis. Obes. Facts.

[25] Knauss C., Paxton S.J., Alsaker F.D. (2007). Relationships amongst body dissatisfaction, internalisation of the media body ideal and perceived pressure from media in adolescent girls and boys. Body Image.

[26] Cash T.F., Pruzinsky T. (2008). Body Image: A Handbook of Theory, Research, and Clinical Practice.

[27] Powell E., Wang-Hall J., Bannister J.A., Colera E., Lopez F.G. (2018). Attachment security and social comparisons as predictors of Pinterest users’ body image concerns. Comput. Human. Behav..

[28] Festinger L. (1954). A theory of social comparison processes. Hum. Relat..

[29] Neumark-Sztainer D., Wall M., Larson N.I., Eisenberg M.E., Loth K. (2011). Dieting and disordered eating behaviors from adolescence to young adulthood: Findings from a 10-year longitudinal study. J. Am. Diet. Assoc..

[30] Aparicio-Martinez P., Perea-Moreno A.J., Martinez-Jimenez M.P., Redel-Macías M.D., Pagliari C., Vaquero-Abellan M. (2019). Social Media, Thin-Ideal, Body Dissatisfaction and Disordered Eating Attitudes: An Exploratory Analysis. Int. J. Environ. Res. Public Health.

[31] Perloff R.M. (2014). Social Media Effects on Young Women’s Body Image Concerns: Theoretical Perspectives and an Agenda for Research. Sex Roles.

[32] Moorhead S.A., Hazlett D.E., Harrison L., Carroll J.K., Irwin A., Hoving C. (2013). A New Dimension of Health Care: Systematic Review of the Uses, Benefits, and Limitations of Social Media for Health Communication. J. Med. Internet Res..

[33] Whalen R., Harrold J., Child S., Halford J., Boyland E. (2018). The Health Halo Trend in UK Television Food Advertising Viewed by Children: The Rise of Implicit and Explicit Health Messaging in the Promotion of Unhealthy Foods. Int. J. Environ. Res. Public Health.

[34] Kotov R.I., Bellman S.B., Watson D.B. (2004). Multidimensional Iowa Suggestibility Scale (MISS) Brief Manual. https://dspace.sunyconnect.suny.edu/handle/1951/60894.

[35] Maraldo T.M., Zhou W., Dowling J., Vander Wal J.S. (2016). Replication and extension of the dual pathway model of disordered eating: The role of fear of negative evaluation, suggestibility, rumination, and self-compassion. Eat. Behav..

[36] Fadul Y.M., Assistance M., Alsanosi A., Hassan Y., Mohammed Z.O., Elhaj A. (2022). Body image and its relationship to suggestibility for students of Alsalam University. Rashhat-e-Qalam.

[37] Mask L., Blanchard C.M. (2011). The protective role of general self-determination against ‘thin ideal’ media exposure on women’s body image and eating-related concerns. J. Health Psychol..

[38] Ray M.K., Zachmann A.E., Caudill C.V., Boggiano M.M. (2020). Relationship between trait suggestibility and eating-related behaviors in overweight and obesity. Eat. Behav..

[39] Deyoub P.L. (1978). Relation of suggestibility to obesity. Psychol. Rep..

[40] Elosua P., López-Jaúregui A., Sánchez-Sánchez F. (2010). EDI-3, Inventario de Trastornos de la Conducta Alimentaria-3, Manual.

[41] Garner D.M. (2004). Eating Disorder Inventory–3 Professional Manual.

[42] Elosua P., López-Jáuregui A. (2012). Internal Structure of the Spanish Adaptation of the Eating Disorder Inventory-3. Eur. J. Psychol. Assess..

[43] Gonzalez-Ordi H., Miguel-Tobal J.J. (1999). Características de la sugestionabilidad y su relación con otras variables psicológicas [Characteristics of suggestibility and its relationship with other psychological variables]. Ann. Psychol..

[44] Hayes A.F. (2022). Introduction to Mediation, Moderation, and Conditional Process Analysis: A Regression-Based Approach.

[45] Li X., Wong W., Lamoureux E.L., Wong T.Y. (2012). Are Linear Regression Techniques Appropriate for Analysis When the Dependent (Outcome) Variable Is Not Normally Distributed?. Investig. Ophthalmol. Vis. Sci..

[46] Fuller-Tyszkiewicz M., Chhouk J., McCann L.A., Urbina G., Vuo H., Krug I., Ricciardelli L., Linardon J., Broadbent J., Heron K. (2019). Appearance comparison and other appearance-related influences on body dissatisfaction in everyday life. Body Image.

[47] Laporta-Herrero I., Jáuregui-Lobera I., Barajas-Iglesias B., Santed-Germán M.Á. (2018). Body dissatisfaction in adolescents with eating disorders. Eat. Weight Disord..

[48] McLean S.A., Rodgers R.F., Slater A., Jarman H.K., Gordon C.S., Paxton S.J. (2022). Clinically significant body dissatisfaction: Prevalence and association with depressive symptoms in adolescent boys and girls. Eur. Child Adolesc. Psychiatry.

[49] Bully P., Elosua P. (2011). Changes in Body Dissatisfaction Relative to Gender and Age: The Modulating Character of BMI. Span. J. Psychol..

[50] Dye H. (2015). Are there differences in gender, race, and age regarding body dissatisfaction?. J. Hum. Behav. Soc. Environ..

[51] Basabas M.C., Greaves L., Barlow F.K., Sibley C.G. (2019). Sexual Orientation Moderates the Effect of Gender on Body Satisfaction: Results From a National Probability Sample. J. Sex Res..

[52] Stice E., Nemeroff C., Shaw H.E. (1996). Test of the dual pathway model of bulimia nervosa: Evidence for dietary restraint and affect regulation mechanisms. J. Soc. Clin. Psychol..

[53] Pearl R.L., Puhl R.M. (2014). Measuring internalized weight attitudes across body weight categories: Validation of the Modified Weight Bias Internalization Scale. Body Image.

