# Supplementary material for: The Moderating Effect of Suggestibility on the Relationship between Body Mass Index and Body Dissatisfaction in Women
Source: J Clin Med. 2024 Aug 8;13(16):4647. doi: 10.3390/jcm13164647 (PMC11354597; doi:10.3390/jcm13164647)
Supplement: Supplementary file 1 [file jcm-13-04647-s001.zip › S1 - EDI-3 Body Dissatisfaction questionnaire.pdf]

S1 - EDI-3 Body Dissatisfaction Questionnaire

Extracted from Garner (2004)

Below, you will find a series of phrases that can refer to food and others to feelings or attitudes you experience. Your task is to rate from 0 to 5 the frequency with which these situations occur in your daily life, according to the following scale:

0	NEVER
1	RARELY
2	SOMETIMES
3	OFTEN
4	ALMOST ALWAYS
5	ALWAYS

Answer as honestly as possible. Thank you for your collaboration.

1. I think that my stomach is too big	0	1	2	3	4	5
2. I think that my thighs are too large	0	1	2	3	4	5
3. I think that my stomach is just the right size *	0	1	2	3	4	5
4. I feel satisfied with the shape of my body *	0	1	2	3	4	5
5. I like the shape of my buttocks *	0	1	2	3	4	5
6. I think my hips are too big	0	1	2	3	4	5
7. I feel bloated after a normal meal.	0	1	2	3	4	5
8. I think that my thighs are just the right size *	0	1	2	3	4	5
9. I think my buttocks are too large	0	1	2	3	4	5
10. I think that my hips are just the right size *	0	1	2	3	4	5

Score:

Items 1, 2, 6, 7, 9: direct punctuation: Never: 0, Rarely: 0, Sometimes: 1, Often: 2, Almost always: 3, Always: 4

Items 3, 4, 5, 8, 10: inverse punctuation (*): Never: 4, Rarely: 3, Sometimes: 2, Often: 1, Almost always: 0, Always: 0

Body Dissatisfaction = sum of all items' punctuations.

Reference

Garner, D.M. *Eating Disorder Inventory– 3 Professional manual*. Odessa, Fl: Psychological Assessment Resources; 2004.
