# Supplementary material for: The Moderating Effect of Suggestibility on the Relationship between Body Mass Index and Body Dissatisfaction in Women
Source: J Clin Med. 2024 Aug 8;13(16):4647. doi: 10.3390/jcm13164647 (PMC11354597; doi:10.3390/jcm13164647)
Supplement: Supplementary file 1 [file jcm-13-04647-s001.zip › S2 - Suggestibility Inventory.pdf]

S2 - Suggestibility Inventory

Translated from Spanish version by Gonzalez-Ordi & Miguel-Tobal (1999)

Below, you will find a series of phrases that can refer to your way of being or reacting to everyday life situations. Your task is to rate from 0 to 4 the frequency with which these situations occur in your daily life, according to the following scale:

0	ALMOST NEVER
1	RARELY
2	SOMETIMES YES SOMETIMES NO
3	MANY TIMES
4	ALMOST ALWAYS

Answer as honestly as possible. Thank you for your collaboration.

**1.**	The opinions of others mean a lot to me.	0	1	2	3	4
**2.**	I can imagine things clearly and vividly.	0	1	2	3	4
**3.**	I am easily influenced by others.	0	1	2	3	4
**4.**	It is easy for me to be affected by the moods of others.	0	1	2	3	4
**5.**	I am a suggestible person.	0	1	2	3	4
**6.**	I have superstitious ideas or thoughts.	0	1	2	3	4
**7.**	I am often affected by horror movies.	0	1	2	3	4
**8.**	When I focus on something, I can become absorbed in it.	0	1	2	3	4
**9.**	I am an impressionable person.	0	1	2	3	4
**10.**	When I focus on my thoughts, I easily lose track of time.	0	1	2	3	4
**11.**	When I listen to music I like, I get carried away by it to the point that I forget about everything else.	0	1	2	3	4
**12.**	The sound of a voice can be so fascinating to me that I can continue listening to it and forget about my surroundings.	0	1	2	3	4
**13.**	I have an easy ability to concentrate on the task at hand.	0	1	2	3	4
**14.**	While watching a movie, TV show, or play, I can become so involved that I forget myself and my surroundings, and experience the plot as if it were real or I were a part of it.	0	1	2	3	4
**15.**	I can recall certain past experiences in my life with such clarity and realism that it is as if I am living them again.	0	1	2	3	4
**16.**	I fantasize about things that happen to me or that I would like to happen to me.	0	1	2	3	4
**17.**	I daydream.	0	1	2	3	4
**18.**	Emotional movies make me cry easily.	0	1	2	3	4
**19.**	I change my mind easily.	0	1	2	3	4
**20.**	I tend to believe what others tell me.	0	1	2	3	4
**21.**	There are people who fascinate me at first sight.	0	1	2	3	4
**22.**	I let certain people influence me more than I think is appropriate.	0	1	2	3	4

<u>Score:</u>

Almost Never: 0, Rarely: 1, Sometimes yes sometimes no: 2, Many times: 3, Almost always: 4.

Dreaming/fantasy subscale: Sum of items 11, 12, 16 and 17 punctuations

Absorption subscale: Sum of items 2, 8, 13 and 15 punctuations

Emotional involvement subscale: Sum of items 6, 7, 9, 14 and 18 punctuations

Influencing-by-others subscale: Sum of items 1, 3, 4, 20 and 22 punctuations

Suggestibility General Index: Sum of all items' punctuations.

Reference

Gonzalez-Ordi, H.; Miguel-Tobal, J.J. Características de la sugestionabilidad y su relación con otras variables psicológicas [Characteristics of suggestibility and its relationship with other psychological variables]. *An Psicol.* **1999**, *15*, 57–75. Available from: https://www.redalyc.org/articulo.oa?id=16715105
